# Comparison of clinical features and prognosis between ultrashort-segment and short-segment hirschsprung disease

**DOI:** 10.3389/fped.2022.1061064

**Published:** 2023-01-06

**Authors:** Chuanping Xie, Jiayu Yan, Jianlin Guo, Yakun Liu, Yajun Chen

**Affiliations:** ^1^ Department of General Surgery, Beijing Children's Hospital, Capital Medical University, National Center for Children's Health, Beijing, China; ^2^ Department of Radiology, Beijing Children's Hospital, Capital Medical University, National Center for Children's Health, Beijing, China

**Keywords:** ultrashort-segment hirschsprung disease, transanal endorectal pullthrough, complications, bowel function outcomes, clinical features

## Abstract

**Objective:**

To compare the differences in clinical features, postoperative complications, and long-term bowel function outcomes of ultrashort-segment Hirschsprung disease (USHD) and short-segment Hirschsprung disease (SHD).

**Methods:**

A retrospective study was conducted to compare patients with USHD or SHD who underwent transanal endorectal pull-through (TEPT) at Beijing Children's Hospital between January 2014 and June 2021. Clinical details were collected from medical records. A long-term bowel function questionnaire (age > 4 years old) was completed by the patients' parents.

**Results:**

A total of 84 patients (USHD = 15, SHD = 69) were included. Age at diagnosis and radical surgery in the USHD group were significantly older than the SHD group (46 [38, 66] vs. 34 [6, 55] months, *p* = 0.002; 51 [39, 68] vs. 37 [10, 68] months, *p* = 0.001, respectively). Compared with the SHD group, patients with USHD are more likely to suffer anastomosis leakage and postoperative enterocolitis after TEPT ([3/15, 33.3%] vs. [1/69, 1.4%], *p* = 0.017; [5/15, 33.3%] vs. [6/69, 8.7%], *p* = 0.023). In addition, patients in the USHD group are inclined to suffer lower bowel function scores (12.0 [7.5, 18.3] vs. 17 [15, 19], *p* = 0.018).Patients in the USHD group were more likely to suffer poorer ability to hold back defecation (*p* = 0.023), soiling (*p* = 0.011), fecal accidents (*p* = 0.004), and social problems (*p* = 0.004).

**Conclusion:**

Compared with patients with SHD, patients with USHD are diagnosed and performed TEPT at an older age. and they are inclined to suffer postoperative enterocolitis, anastomosis leakage, and poorer long-term bowel function following TEPT.

## Highlights

• Compared with short-segment Hirschsprung disease (SHD), ultrashort-segment Hirschsprung disease (USHD) was diagnosed and performed radical surgery at an older age.
• USHD are inclined to suffer anastomosis leakage and enterocolitis after TEPT.
• USHD are inclined to suffer anastomosis leakage and enterocolitis after TEPT.

## Introduction

Hirschsprung disease (HD), known as aganglionosis, is one of the most common congenital malformations, with an incidence of 1 in 5,000 live births (1, 2). More than 80% of HD patients had aganglionosis restricted to the rectum and sigmoid colon. However, there is minimal knowledge concerning ultrashort-segment HD (USHD), a rare variant of HD (3). So far, there has been no universally accepted definition for USHD. Some scholars considered USHD and internal anal sphincter achalasia (IASA) as the same entity, which could be alleviated by sphincter myotomy or botulinum toxin (4, 5). A recent study proposed that they were utterly different diseases (6, 7). IASA was characterized by normal ganglion cells in the rectal mucosal biopsy but the absence of nitrergic innervation or defective innervation of the neuromuscular junction, resulting in motility dysfunction. Anal sphincter myotomy or botulinum toxin might be the primary treatment (4, 7, 8). However, USHD, presenting as a suddenly dilated bowel without an obvious transition zone on barium enema, is characterized by aganglionosis extending proximal to the distal rectum, and transanal endorectal pull-through (TEPT) might be required (3, 7, 9). In our study, we chose the latter to define USHD.

In recent years, postoperative complications and long-term bowel function recovery of HD has become gradually attracted attention (10, 11). Previous studies had revealed the postoperative complications and long-term bowel function operated by Soave, Duhamel, or Swenson (12, 13). However, few studies were concerned about the treatment and prognosis of different types of HD, especially for patients with USHD. This study was designed to compare the differences in clinical features, postoperative complications, and long-term bowel function outcomes between USHD and SHD following TEPT, aiming to provide guidance for the diagnosis and treatment of USHD.

## Materials and methods

### Patient selection

Approved by the Ethics Committee of Beijing Children's Hospital, we reviewed the medical records of 84 consecutive patients with rectosigmoid HD who underwent primary TEPT at Beijing Children's Hospital, National Center for Children's Health, between January 2014 and June 2021. The operation was performed by either totally transanal performed TEPT (TTEPT) or laparoscopic/laparotomy-assisted TEPT (LTEPT). All patients underwent Soave TEPT without a stoma by the same surgeon team. Patients who underwent radical surgery in other hospitals or patients without a completely preoperative radiography examination were excluded. Rectal biopsies and postoperative histopathological examination verified the diagnosis of HD. Contrast enemas was used to estimate the extension of the aganglionosis segment in all patients.

### Study design

**Figure 1:**
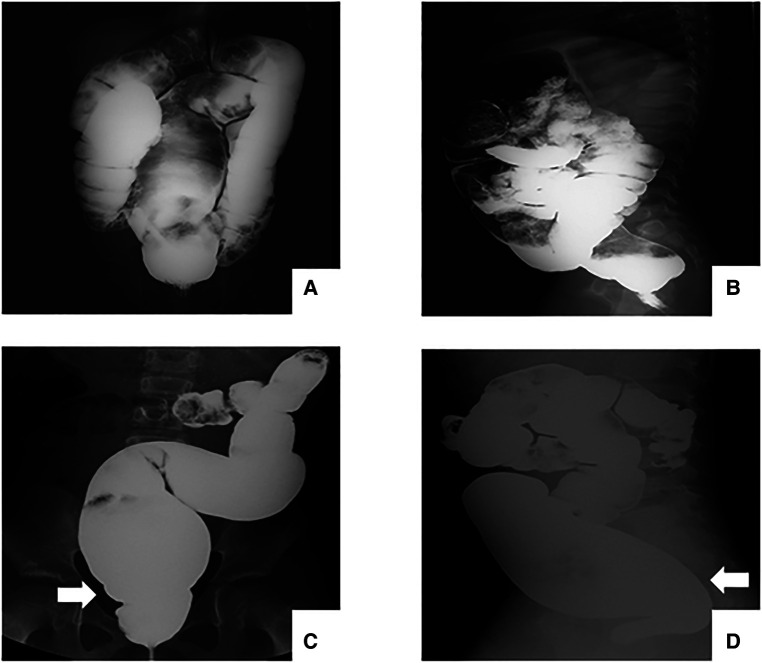
Radiography examination before TEPT. (**A-B**) patients with USHD shows a suddenly dilated bowel without an obvious transition zone. (**C-D**) patients with SHD shows a clear transition zone.

Considering the significant imaging differences between USHD and SHD, the USHD was defined as presenting a suddenly dilated bowel without an obvious transition zone on preoperative barium enema based on the absence of ganglion cells 3–4 cm above the pectinate line by rectal mucosal biopsy. To distinguish the presence of a transitional zone, all preoperative radiographs of HD were independently and randomly reviewed by an experienced pediatric radiologist and two practiced doctors. For the disputed imaging results, these radiography examinations were reviewed again to detect the presence of a transitional zone. Rectosigmoid HD was divided into two groups according to whether a transition zone presented on barium enema: the USHD group was defined as presenting a suddenly dilated bowel without an obvious transition zone (Figures 1A,B), and the short-segment (SHD) group as aganglionosis extending proximal to the rectum and sigmoid with a transition zone (Figures 1C,D). A comparative study was performed to analyze the difference in clinical manifestations, postoperative complications, and long-term bowel function outcomes between the two groups.

### Acquisition of data

The patient's characteristics and clinical details were recorded retrospectively from medical records, including gender, birth weight, gestational age, congenital malformations, age at diagnosis, presenting symptoms, surgical details, and postoperative complications.

Bowel function outcome was evaluated by a bowel function score (BFS) questionnaire established by 7-item scoring systems with a maximum score of 20 (Table 1). Patients' parents filled out all the questionnaires. The BFS was only assessed when the children were older than 4 years old. Patients were also inquired about the history of enterocolitis and other postoperative complications following TEPT. Enterocolitis was diagnosed when patients presented with clinical signs of bowel inflammation, such as abdominal distension, diarrhea, fever, or lethargy.

**Table 1:** Bowel function score questionnaire (>4 years old).

Evaluation of bowel control	Score
Ability to hold back defecation, *n* (%)	
Always	3
Problems less than 1/week	2
Weekly problems	1
No voluntary control	0
Feels/reports the urge to defecate, *n* (%)	
Always	3
Most of the time	2
Uncertain	1
Absent	0
Frequency of defecation, *n* (%)	
Every other day to twice a day	2
More than	1
Less than	1
Soiling, *n* (%)	
Never	3
Staining < 1/week, no change of underwear required	2
Frequent staining, change of underwear often required	1
Daily soiling, requires protective aids	0
Fecal accidents, *n* (%)	
Never	3
Fewer 1/week	2
Weekly, requires protective aids	1
Daily, requires protective aid day and night	0
Constipation, *n* (%)	
No constipation	3
Manageable with diet	2
Manageable with laxatives	1
Manageable with enemas	0
Social problems, *n* (%)	
No social problems	3
Sometimes	2
Problems restricting social life	1
Severe social/psychosocial problems	0

### Statistical analysis

Statistical analysis was conducted using IBM SPSS Statistics for Statistics ver. 26.0 Software. Data were presented as frequency (percentage) for qualitative variables and median and interquartile range (IQR) for continuous variables. All the statistical tests were two-sided, with a significant level of *p* < 0.05. Continuous Chi-squared tests or Fisher's exact tests (Fisher's exact if >25% of cell have expected counts less than 5) were applied for categorical variables. Independent sample t-test or Mann-Whitney U test (Mann-Whitney if the data did not meet normal distribution) for continuous variables.

## Results

### Patient characteristics

**Figure 2:**
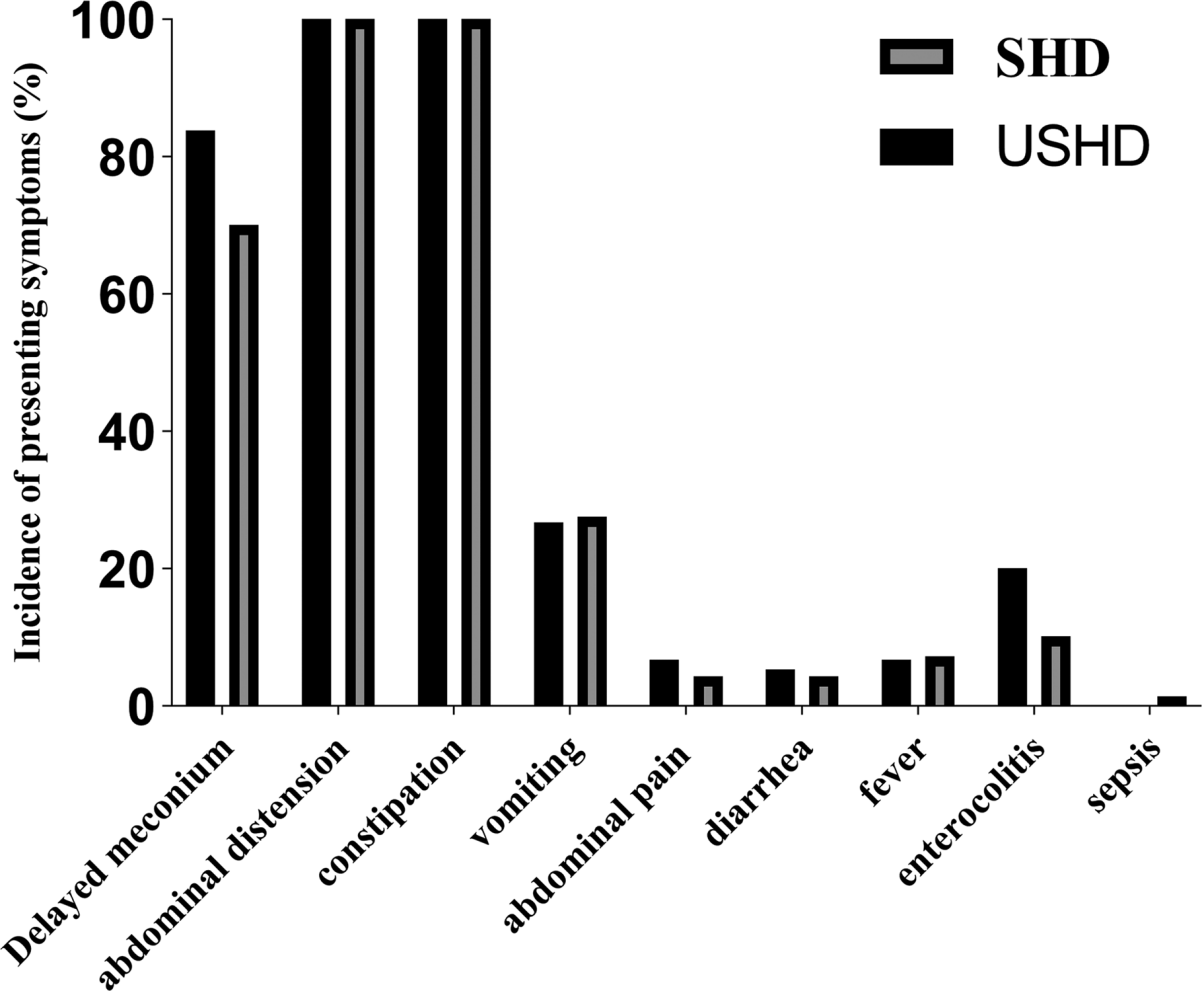
Difference in clinical mainfestaions between USHD and SHD group before admission.

A total of 84 patients were enrolled in our study, including 15 patients with USHD and 69 with SHD. The clinical manifestations of all patients before admission are presented in Figure 2. No significant difference was observed between the two groups. Abdominal pain, constipation, and delayed meconium were the predominant clinical manifestations.

The baseline characteristics of all patients are presented in Table 2. The age at diagnosing HD, age at radical surgery, and weight at radical surgery in the USHD group were significantly higher than in the SHD group, respectively (age at diagnosis, 46 [38, 66] vs. 34 [6, 55] months, *p* = 0.002; age at surgery, 51 [39, 68] vs. 37 [10, 68] months, *p* = 0.001; weight at surgery, 15 [14, 18] vs. 12 [9, 17] Kg, *p *< 0.001). The frequency of TTEPT or LTEPT was similar in both groups. However, there were significantly prolonged hospital days following TEPT in the USHD group despite no statistically significant differences in operation time, blood loss, oral intake time, and hospital days.

**Table 2:** Patient characteristics.

Characteristics	USHD (*n* = 15)	SHD (*n* = 69)	*p*-Value
Sex			
Male	15 (100)	57 (82.6)	0.113
Female	0 (0.0)	12 (17.4)	
Gestational age			
Preterm (<37 weeks)	2 (13.3)	3 (4.3)	0.216
Term (>37 weeks)	13 (86.7)	66 (95.7)	
Birthweight in kilograms, mean (SD)	3.40 ± 0.54	3.25 ± 0.48	0.274
Congenital malformation			
Yes	1 (6.7)	4 (5.8)	>0.999
No	14 (93.3)	65 (94.2)	
Age at diagnosis, months	46 [38, 66]	34 [6, 55]	0.002
Age at radical surgery, months	51 [39, 68]	37 [10, 68]	0.001
Weight at radical surgery, kilogram	15 [14, 18]	12 [9, 17]	<0.001
Surgical approach			
Transanal only	12 (80.0)	51 (73.9)	0.374
Open surgery + transanal	1 (6.7)	13 (18.8)	
Laparoscopic + transanal	2 (13.3)	5 (7.2)	
Operation time, min	130 [98, 240]	108 [80, 154]	0.170
Blood loss, ml	2 [2, 10]	5 [2, 5]	0.929
Oral intake time, days	3 [3, 5]	3 [3, 4]	0.142
Hospital stays in days	12 [9, 15]	14 [9, 20]	0.337
Total length of hospital stays after surgery, days	11 [7, 15]	8 [7, 10]	0.015

Data are presented as median [IQR, interquartile range] and frequency (%).

### Postoperative complications

Table 3 shows postoperative complications between the two groups. All the patients were followed up for more than one year with a median follow-up time of 5.3 years (4.9 [3.1, 6.4] vs. 5.4 [2.9, 6.9], *p* = 0.691). Postoperative complications included anastomotic leakage or stricture, postoperative enterocolitis, and residual transitional zone. Compared with the SHD group, anastomosis leakage after TEPT was more likely to occur in the USHD group ([3/15, 33.3%] vs. [1/69, 1.4%], *p* = 0.017). These patients with anastomosis leakage often presented fever, abdominal pain, abdominal distention, blood stool, and severe infection, and ileostomies and anastomotic resuturing were performed. In addition, the frequency of postoperative enterocolitis in the USHD group was significantly higher than in the SHD group ([5/15, 33.3%] vs. [6/69, 8.7%], *p* = 0.023). No significant difference in both groups was observed in terms of residual transitional zone.

**Table 3:** Postoperative complications.

	USHD (*n* = 15)	SHD (*n* = 69)	*p*-Value
Mean follow-up time, years	4.6 [3.1, 6.4]	5.4 [2.9, 6.9]	0.691
Anastomosis leakage, *n* (%)	3 (20.0)	1 (1.4)	0.017
Enterocolitis, *n* (%)	5 (33.3)	6 (8.7)	0.023
Residual transitional zone, *n* (%)	0 (0.0)	1 (1.4)	>0.999

Data are presented as median [IQR, interquartile range] and frequency (%).

### Long-term bowel function outcomes

**Figure 3:**
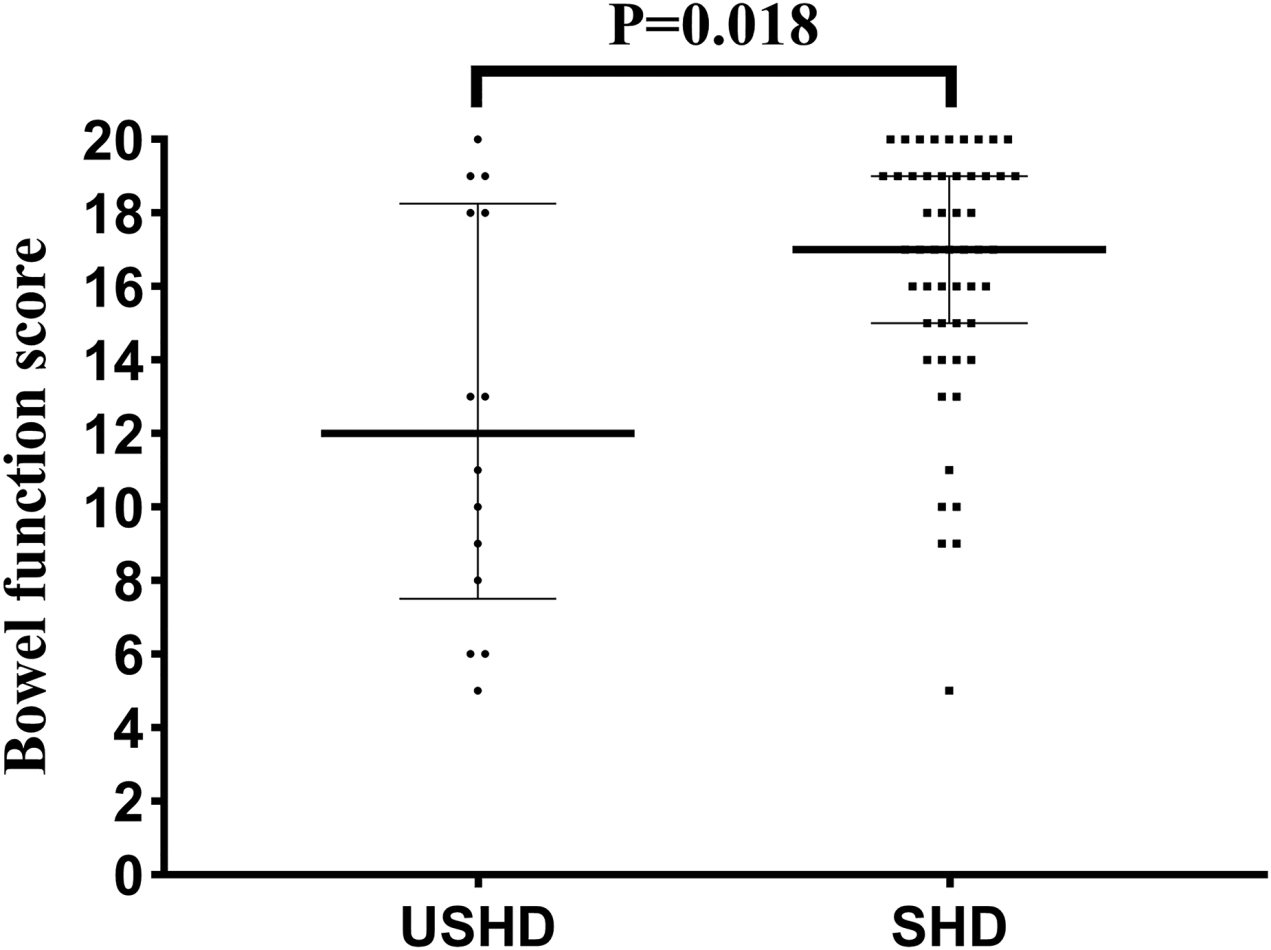
BFS for each patient in the USHD group and the SHD group.

Table 4 presents the results of the BFS questionnaire completed by patients' parents. There was no statistically significant difference between the two groups in terms of follow-up age (8.7 [7.3, 11.9] vs. 7.8 [5.9, 9.4] years, *p* = 0.119). As shown in Figure 3, the median total bowel function score in the USHD group was significantly lower than the SHD group (12.0 [7.5, 18.3] vs. 17 [15, 19], *p* = 0.018). Besides, the percentage of poorer bowel function in the USHD group was significantly higher than SHD group (*p* = 0.01). Further, compared with the SHD group, we found that patients in the USHD group were more likely to suffer poorer ability to hold back defecation (*p* = 0.023), daily soiling (*p* = 0.011), fecal accidents (*p* = 0.004), and social problems (*p* = 0.004). There was no statistically significant difference between the two groups regarding feeling the urge to defecate, frequency of defecation, and constipation.

**Table 4:** Bowel function score (>4 years old).

Evaluation of bowel control	Score	USHD^a^ (*n* = 14)	SHD^b^ (*n* = 52)	*p*-Value
Mean follow-up age, years		8.7 [7.3, 11.7]	7.8 [5.9, 9.4]	0.119
Ability to hold back defecation, *n* (%)				
Always	3	5 (35.7)	31 (59.6)	0.023
Problems less than 1/week	2	1 (7.1)	12 (23.1)	
Weekly problems	1	3 (21.4)	4 (7.7)	
No voluntary control	0	5 (35.7)	5 (9.6)	
Feels/reports the urge to defecate, *n* (%)				
Always	3	5 (35.7)	28 (52.8)	0.089
Most of the time	2	3 (21.4)	16 (30.2)	
Uncertain	1	2 (14.3)	6 (11.3)	
Absent	0	4 (28.6)	3 (5.7)	
Frequency of defecation, *n* (%)				
Every other day to twice a day	2	10 (71.4)	34 (65.4)	>0.999
More than	1	3 (21.4)	14 (26.9)	
Less than	1	1 (7.1)	4 (7.7)	
Soiling, *n* (%)				
Never	3	2 (14.3)	17 (32.7)	0.011
Staining < 1/week, no change of underwear required	2	3 (21.4)	17 (32.7)	
Frequent staining, change of underwear often required	1	3 (21.4)	15 (28.8)	
Daily soiling, requires protective aids	0	6 (42.9)	3 (5.8)	
Fecal accidents, *n* (%)				
Never	3	5 (35.7)	39 (75.0)	0.004
Fewer 1/week	2	1 (7.1)	7 (13.5)	
Weekly, requires protective aids	1	6 (42.9)	4 (7.7)	
Daily, requires protective aid day and night	0	2 (14.3)	2 (3.8)	
Constipation, *n* (%)				
No constipation	3	11 (78.6)	44 (84.6)	0.053
Manageable with diet	2	0 (0.0)	6 (11.5)	
Manageable with laxatives	1	3 (21.4)	2 (3.8)	
Manageable with enemas	0	0 (0.0)	0 (0.0)	
Social problems, *n* (%)				
No social problems	3	8 (57.1)	44 (84.6)	0.004
Sometimes	2	5 (35.7)	3 (5.8)	
Problems restricting social life	1	0 (0.0)	3 (9.6)	
Severe social/ psychosocial problems	0	1 (7.1)	0 (0.0)	
Total BFS, *n* (%)				
Good bowel function	≥17	5 (35.7)	30 (57.7)	0.010
Moderate bowel function	12–16	2 (14.3)	16 (30.8)	
Poor bowel function	<12	7 (50.0)	6 (11.5)	

Data are presented as median [IQR, interquartile range] and frequency (%).

^a^ USHD: one patient was excluded due to the loss of follow-up (1/15, 6.7%).

^b^ SHD: seventeen patients were excluded due to the loss of follow-up (11/69, 15.9%) and less than 4 years old at follow-up (6/69, 8.7%).

## Discussion

This is the first single-center retrospective study to show that patients with USHD were diagnosed and performed TEPT at an older age, mainly related to non-specific symptoms. We have also shown that anastomosis leakage and postoperative enterocolitis are more likely to occur in the USHD group rather than SHD group after TEPT. In addition, our results suggest that compared with SHD, a higher proportion of patients with USHD may be predisposed to suffering long-term bowel functional defects.

Our study found that the age at diagnosis and radical surgery in both groups was significantly higher than in previous literature reports (14, 15). It might be related to the fact that most patients with HD in our study did not initially develop serious clinical symptoms, and most of them improved after conservative treatments (glycerine enema, polyethylene glycol, et al.) at local hospitals. Most of them would not be transferred to our center for radical surgery until presenting more severe symptoms. Moreover, the age at diagnosing HD and performing radical surgery in the USHD group was significantly older than in the SHD group. This condition was mainly due to the fact that patients with shorter-segment aganglionosis were more likely to relieve the obstructive symptoms by conservation treatment (3, 9, 16). However, the older age at performing radical surgery was an important factor for more severe dilated colon, leading to a higher frequency of early postoperative complications, which could also explain prolonged hospitalization following TEPT in the USHD group (17, 18).

Regarding postoperative complications, the most striking difference in terms of short-term postoperative complications between the USHD group and the SHD group was the incidence of anastomotic leakage. The frequency of anastomosis leakage in the USHD group (20%) was significantly higher than in the SHD group (1.4%). A plausible explanation for this phenomenon might be related to problems of a severely dilated distal rectum (17, 19). It could significantly contribute to the difficulty in dissection during the dissociation above the dentate line and hemodynamic disorder in the distal bowel, leading to a high incidence of anastomosis leakage (17, 20, 21). Some studies even recommended a preoperative stroma to decompress the distal colon to prevent anastomosis leakage (17, 18). Unlike previous studies, our study did not observe anastomotic stricture following TEPT, which might be related to regular anal dilatation two weeks after radical surgery (22, 23). Enterocolitis is the most common postoperative complication. The incidence of postoperative enterocolitis ranged from 10% to 44% based on heterogeneity in case definitions and geographical differences (24–26). However, the incidence of postoperative HAEC in the USHD group (33.3%) was significantly higher than in the SHD group (8.7%). A possible explanation was related to the higher preoperative enterocolitis frequency in the USHD group (26.7% vs. 13.0%) (15, 26). Preoperative enterocolitis episodes could change the gut microbial system, making patients susceptible to the development of further episodes of enterocolitis (27, 28).

Compared with the SHD group, the patients in the USHD group were inclined to suffer lower bowel function scores, and the percentage of poor bowel function in the USHD group (50%) was significantly higher than in the SHD group (11.5%). After careful analysis of the bowel function of these patients with low scores, we found that fecal incontinence was the main reason for poorer bowel function outcomes. Fecal incontinence, which refers to the poor ability to hold back defecation, daily soiling, and fecal accidents, is the most common problem following pull-through, predisposing children to impaired social functioning and emotional psychosocial well-being (11, 29, 30). The reason could explain the high frequency of fecal incontinence that more prolonged and extensive anal dilatation was required due to a severely dilated bowel below the peritoneal reflection, resulting in excessive stretching of the sphincter during TEPT (29, 31–33). To minimize the damage to the anal sphincter, some studies also advocated LTEPT as the primary treatment for HD rather than TTEPT (11, 29, 33). A previous study found internal anal sphincter defects occurred more often following TEPT by endosonography (32). However, recent studies did not find a significant difference in long-term bowel function outcomes between the two surgical approaches (34,35). In our study, three patients in the USHD group who underwent laparoscopic/laparotomic-assisted TEPT did not achieve good long-term bowel function. The reason was that the dissection of the severely dilated distal rectum was equally tricky whether choosing LTEPT or TTEPT.

It was the first study to identify that USHD was prone to suffering anastomosis leakage, postoperative enterocolitis, and poorer long-term bowel function outcomes than SHD. In addition, all the TEPT procedures were performed by the same surgeon, avoiding deviation due to the discrepancy in surgical details. However, this study has some limitations. First and foremost, there is no universally accepted definition for USHD. In our study, the USHD was defined as s presenting a suddenly dilated bowel without an obvious transition zone without transitional zone on preoperative barium enema based on the absence of ganglion cells of the distal rectum by rectal mucosal biopsy and postoperative histopathology examination. Second, a small number of cases of the USHD group might lead to a potential selection bias. Third, this study was retrospective in nature and included patients treated at a single center, leading to a particular deviation. Last but not least, despite a dropout of 16.7%, it remained unknown whether their bowel function result differed in those who completed and those who did not.

## Conclusion

In conclusion, our study suggests that compared with SHD, USHD presenting as a suddenly dilated bowel without an obvious transition zone on barium enema has a greater delay in the age of diagnosis and radical surgery. After TEPT, USHD is more likely to suffer anastomosis leakage, enterocolitis, and poor long-term bowel function outcome, which was related to the problem of a severely distal dilated rectum. For USHD, it is a therapeutic challenge for pediatric surgeons to enhance earlier diagnosis rates and improve long-term bowel function following TEPT.

## Data Availability

The raw data supporting the conclusions of this article will be made available by the authors, without undue reservation.
